# The role of reactive enteric glia‐macrophage interactions in acute and chronic inflammation

**DOI:** 10.1111/nmo.14947

**Published:** 2024-10-20

**Authors:** Schneider Reiner, Schneider Linda, Hamza Ebrahim, Leven Patrick, Wehner Sven

**Affiliations:** ^1^ Department of Surgery University Hospital Bonn Bonn Germany

**Keywords:** enteric glia, enteric nervous system, intestinal inflammation

## Abstract

Enteric glia are a heterogeneous population of peripheral glia within the enteric nervous system and play pivotal roles in gut homeostasis, tissue integrity, coordination of motility, and intestinal immune responses. Under physiological conditions, they communicate with enteric neurons to control intestinal motility. In contrast, enteric glia undergo reactive changes in response to inflammatory signals during enteric neuroinflammation and participate in immune control. In this state, these glia are called reactive enteric glia, which promote cytokine and chemokine secretion and perpetuate immune cell recruitment, thereby affecting disease progression. Interestingly, reactive glia exhibit a huge plasticity and adapt to or shape the immune environment towards a resolving phenotype during inflammation and neuropathies. Recent studies revealed a bidirectional communication between enteric glia and resident and infiltrating immune cells under healthy conditions and in the context of inflammation‐based intestinal disorders and neuropathies. While recent reviews give a superb general overview of enteric glial reactivity, we herein discuss the latest evidence on enteric glial reactivity in two prominent inflammatory conditions: acute postoperative inflammation, resulting in postoperative ileus, and chronic inflammation in inflammatory bowel diseases. We define their plasticity during inflammation and the interplay between reactive enteric glia and intestinal macrophages. Finally, we sketch important questions that should be addressed to clarify further the impact of enteric glial reactivity on intestinal inflammation.

**Key points**

- Enteric glia play a crucial role in regulating GI motility through Ca^2+^ signaling and bidirectional communication with neurons, transitioning to a reactive state during pathophysiological conditions, contributing to neuroinflammation.
- Reactive enteric glia play a significant role in POI by releasing cytokines and chemokines in response to inflammation, surgical trauma, and adrenergic signaling, contributing to immune modulation, macrophage recruitment, and glial reactivity.
- In chronic intestinal inflammation, reactive enteric glia exacerbate neuroinflammation through interactions with immune cells, contributing to neuronal death, visceral pain, and the progression of inflammation.

## 1 INTRODUCTION

Enteric glia are specialized cells of the enteric nervous system (ENS) that contribute to intestinal homeostasis and disease states.^1^ A wealth of information exists on the role of enteric glia in maintaining tissue integrity and barrier function.^2^, ^3^, ^4^, ^5^ Different glial cell populations add to the generalized view of enteric glia as a crucial component of a functional gut, and the current knowledge describes local, regional, and functional heterogeneity between enteric glial subpopulations.^1^, ^6^ Local heterogeneity refers to differences among glia within a given region of the gastrointestinal tract.^7^ Transcriptional studies at the single‐cell level during homeostasis revealed six^8^ or seven^9^ types of enteric glia in humans or mice, respectively. However, others only found two clusters of EGCs in mice, of which only one corresponded to a distinct reactive phenotype.^10^ While further studies are needed to confirm the existence of phenotypically and functionally different enteric glia populations, the current knowledge supports a certain degree of heterogeneity and difference in their reactivity.^11^ The occurring characteristics of “activated” and “reactive” glial states have been summarized recently.^1^, ^10^, ^11^, ^12^, ^13^ The differences in glial transcription patterns also indicate differences along the GI tract and across the layers of the intestinal wall, as well as their localization within or outside of enteric ganglia.^14^ These subtypes include glia associated with neuronal cell bodies in the myenteric and submucosal plexuses, indicating communication with enteric neurons to control various ENS functions, which include gastrointestinal motility and secretomotor activity^3^, ^15^ as major tasks of enteric glia.^16^ The prominence of enteric glia in the ENS is also highlighted by their amount, as they outnumber enteric neurons in many species,^17^, ^18^, ^19^ with up to five times more glia than neurons.^12^, ^19^

Furthermore, enteric glia are known for their neurogenic potential upon damage to the ENS.^20^, ^21^, ^22^ However, how crucial the role of glial neurogenesis is remains still unclear. The loss of or damage to enteric neurons is prevalent in many pathologies and is often summarized as neuropathies. These are present in chronic intestinal pseudo‐obstructions, constipation, and irritable bowel syndrome,^23^ but also include inflammation‐driven disorders, such as postoperative ileus (POI) and inflammatory bowel disease (IBD), that are accompanied by major functional abnormalities in the ENS. Given their involvement in congenital or acquired neuropathies, enteric glia have been implied as a potential target in treating ENS neurodegeneration.^24^, ^25^

**FIGURE 1:**
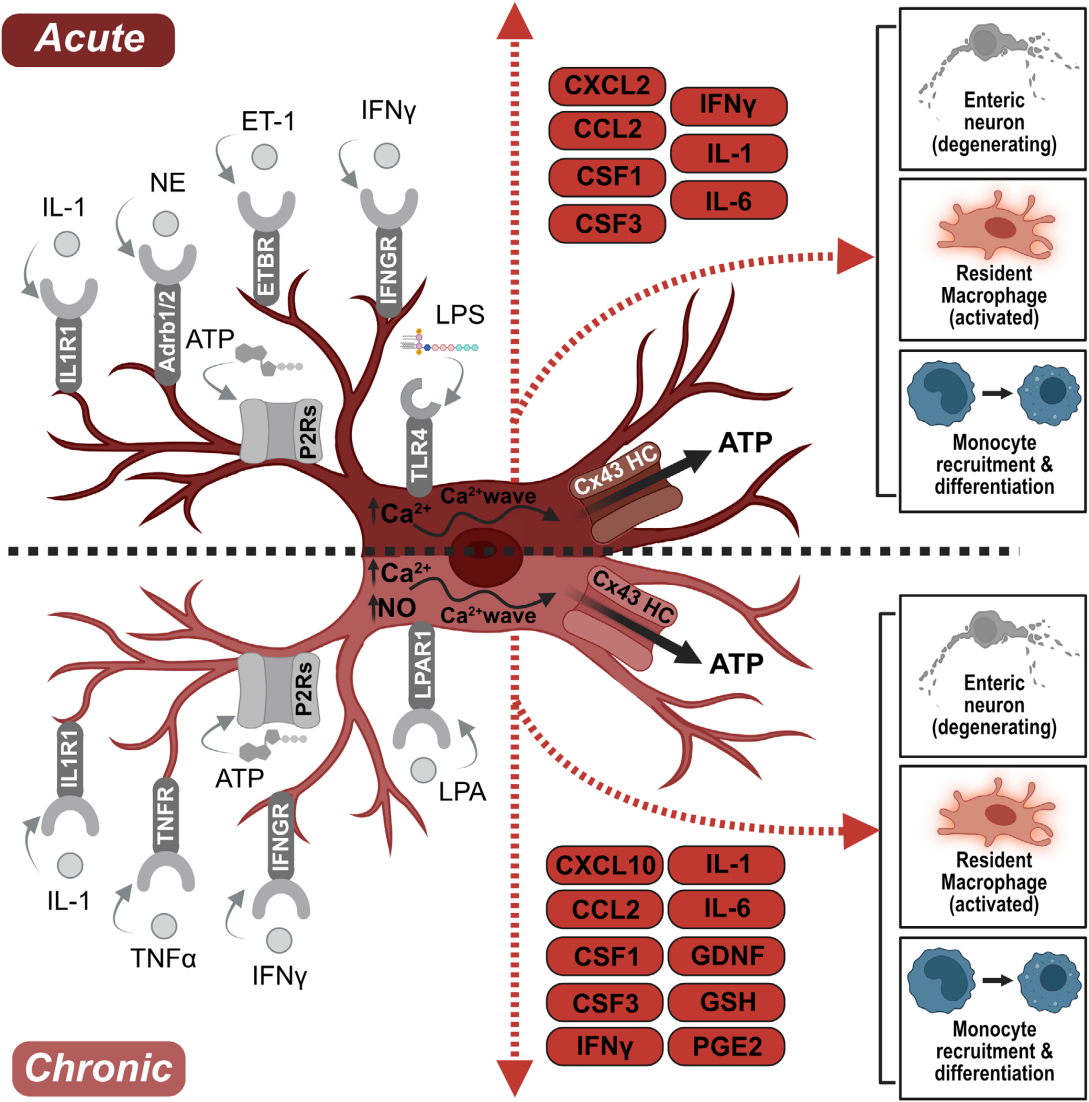
Schematic representation of reactive glia in acute and chronic conditions. Activators (gray, left side) acting on specific receptors on enteric glia (IL1R1, Adrb1 and Adrb2, ETBR, TNFR, IFNGR, TLR4, LPAR1, and different P2Rs (P2XR/P2YR)) and released mediators (red, right side). Increase of glial NO (chronic) and Ca^2+^ (both) followed by Ca^2+^ waves, Cx43 hemichannel opening, and ATP release. Reactive glia‐released mediators (red dotted arrows) act on the surrounding tissue and induce, among others, enteric neuron degeneration, resident macrophage activation, and monocyte recruitment and differentiation. This figure was created with biorender.com.

Within the last years, enteric glia have been shown to interact not only with enteric neurons but also with various other cell types (reviewed in^1^), including extrinsic neurons,^16^ epithelial cells,^2^ epithelial stem cells, and mesenchymal cells,^26^ and especially, immune cells.^27^ Consequently, enteric glia became a focus in inflammation‐related disorders and diseases due to their ability to acquire a so‐called “reactive” phenotype, wherein they undergo morphological, transcriptomic, and functional changes and control local inflammation in damaged or acute and chronically inflamed tissue.^1^, ^28^ Depending on their activation state, the surrounding environment, and the inflammatory context, enteric glia can exert either detrimental^29^, ^30^ or beneficial actions^31^, ^32^, ^33^, ^34^ on the intestinal tissue.^1^ A nonexclusive overview of these pathologies linked to reactive enteric glia and involved mediators is given in Table 1 and Figure 1.

**TABLE 1:** A nonexclusive list of neuropathic diseases and disorders and recently identified interacting partners, activators, and released mediators of reactive enteric glia.

Disease	Interacting cells	Activators	Glial transcriptional changes	Functional validation	Outcome	Ref.
POI	EN	exATP on P2X2	Cxcl2, Il‐6	Pharmacological intervention	GI‐Transit ↓, Immune infiltration ↑	^35^
POI	rMM	IL‐1β	Il‐6, Ccl2, Csf1, Csf3	Transgenic intervention	GI‐Transit ↓, Immune infiltration ↑	^36^
POI	SN	NE	Il‐6, Ccl2	Transgenic intervention	GI‐Transit ↓, Immune infiltration ↑	^37^
POI	EN	ET‐1 on ET_B_R	None Specified	Pharmacological intervention	GI‐Transit ↓, Inflammation ↑, Ca^2+^ modulation	^38^
POI	MC	IL‐1β	Il‐6, Ccl2	Transgenic intervention	GI‐Transit ↓, Immune infiltration ↑	^39^
POI	MC	IL‐1β	Ccl2, Csf‐1	Transgenic intervention	Anti‐inflammatory differentiation ↑	^40^
Colitis	rMM	Pro‐infl. on Cx43	None Specified	Transgenic intervention	Visceral Hypersensitivity ↑, CSF‐1 mediated Immune infiltration/activation ↑	^41^
Colitis	EN	IL‐1β on Cx43	None Specified	Transgenic intervention	PGE2‐mediated Visceral Hypersensitivity ↑	^42^
Colitis	EN & rMM	IL‐1β/α, TNFα	None Specified	Pharmacological intervention	Enteric neuroinflammation and degeneration ↑	^43^
Colitis	EN	None Specified	None Specified	Pharmacological intervention	Glial GSH mediated inflammation, neurodegeneration, and disease worsening	^32^
Colitis	EN	None Specified	None Specified	Transgenic and pharmacological intervention	Glial ATP‐mediated neuronal death coinciding with oxidative stress	^29^
Colitis	EN	ATP on Entpd2	None Specified	Transgenic intervention	Glial Entpd2 mediated disease improvement during acute phases	^34^
Colitis	EN	None Specified	Extensive panel	None	Inflammation triggers a transient, local change in the neuroglial transcriptome	^44^
Inflammation	n/a	LPS, IFNγ	Extensive panel	Ca^2+^ response and ELISA	LPS‐induced dysregulation of glial transcriptome and disruption of purinergic signaling and Ca^2+^ responses	^45^
Infection	FB & IC	IFNγ	Cxcl10	Transgenic intervention	IFNγ triggered EGCs calibrate local immune responses and promote tissue repair	^10^
Oxidative stress	EN	H_2_O_2_, DA	None Specified	Transgenic intervention	Glial GSH mediated neuro‐protection and survival ↑	^33^
SIRS	n/a	LPS	Gfap, S100b, IFNγ, IL‐1β, Il‐6, Ccl2	ELISA	LPS‐induced dysregulation of myenteric glial transcriptome and increased release of inflammatory mediators	^46^
CIPO	EN	LPA on LPAR_1_	Lpar_1_	Pharmacological intervention	Glial LPAR_1_ mediated neuronal remodeling, modulation of Ca^2+^ responses, and GI‐Transit ↓	^47^

Abbreviations: EN, enteric neuron; FB, fibroblast; IC, immune cells; MC, monocyte; n/a, not available; rMM, resident muscularis macrophages; SN, sympathetic neuron.

In this mini‐review, we will focus on the latest findings about the mechanisms and molecules switching enteric glia into a reactive state, their activators, and released mediators, as well as their plasticity during different stages of inflammation. Here, we will mainly focus on two commonly studied pathologies in the field. First, an acute postoperative inflammation resulting in postoperative ileus (POI) and, secondly, colitis, representing the research area of IBD. Moreover, we will take a closer look at intercellular communication of enteric glia and macrophages and close this review with, from our perspective, major open questions in the field of reactive enteric glia function in intestinal inflammation.

## 2 PHYSIOLOGY OR PATHOPHYSIOLOGY: TWO STATES OF ACTIVATED OR REACTIVE ENTERIC GLIA

Enteric glia have been identified as essential regulators of intestinal motility during homeostasis.^3^, ^15^, ^31^, ^48^, ^49^ The underlying mechanisms involve the well‐studied induction of Ca^2+^ signaling in enteric glia,^12^ which depends on connexin‐43 (Cx43) hemichannels.^50^ Interfering with glial Ca^2+^ signaling pathways impacts intestinal motility, leading to a prolonged transit time, while stimulating glial Ca^2+^ responses actively promotes GI motility by interacting with neuronal motor circuits.^50^

Under physiological conditions, enteric glia are relatively silent in quiescent periods of tonic inhibition but become periodically activated by certain neurotransmitters, including acetylcholine and purines like ATP.^47^, ^49^, ^51^ This glial activation leads to the release of gliotransmitters, including ATP and GABA, that act on purinergic or GABAergic receptor‐expressing excitatory or inhibitory neurons, thereby coordinating intestinal motility (reviewed in^1^). Moreover, enteric glia can be activated through type 1 lysophosphatidic acid receptor (LPAR1) signaling, where disturbances result in enteric neuro‐ and gliopathy^52^ accompanied by impaired GI motility. Patients suffering from a severe motility disorder and chronic intestinal pseudo‐obstruction (CIPO) had reduced glial LPAR1 expression in their intestines. Another motility‐related study by Mazzotta et al. showed that enteric glia become activated by endothelin‐1/endothelin receptor B (ET‐1/ETB_R_) signaling and subsequently inhibit excitatory cholinergic neurons and stimulate inhibitory nitrergic motor pathways thereby impeding motility.^38^ The fundamental role of the bidirectional communication between enteric neurons and enteric glia has recently been reviewed,^1^ and several lines of evidence support the important role of enteric glia in setting the tone of gastrointestinal reflexes. Interestingly, these data show that enteric glia become activated during pathological conditions, sometimes even by the same mediators acting on them under physiological conditions. Enteric glial activation regularly occurs under physiological conditions, while pathological conditions induce a transformation towards a so‐called “reactive” phenotype, which has been summarized recently.^1^ Further findings support the concept that enteric glia are involved in these pathological processes by inflicting neuropathies in the ENS^23^, ^45^, ^53^ and that “reactive glia” contribute to neuroinflammation and abnormal GI motility. This research has led to a better, albeit incomplete, understanding of glial reactivity under pathophysiological conditions.

### 2.1 Reactive enteric glia in acute inflammation

Despite massive technical improvements in surgical procedures, transient motility disturbances occur frequently after abdominal surgery and result in postoperative ileus (POI). POI is defined by transiently reduced motility, nausea, vomiting, and increased morbidity, which leads to extended hospitalization and a high medico‐economic burden.^54^ Consensus about transient inflammation of the *muscularis externa* being the main reason for POI has existed for over a decade.^54^ This inflammation is induced by surgical manipulation/surgical trauma to abdominal/intestinal surfaces, and the subsequent release of inflammatory mediators leads to enteric neuronal dysfunction, disrupted neuronal signaling, and, ultimately, GI motility impairment.

The first evidence of enteric glial involvement in POI pathophysiology came from Stoffels et al., showing that IL‐1β, a cytokine mainly released from infiltrating monocyte‐derived macrophages, induces a “reactive” state in enteric glia.^39^ The latter respond by releasing IL‐6.^39^ Of note, IL‐6 is a complex cytokine released from various cell populations, including enteric neurons, modulating multiple cell types, such as regulatory T‐cells in the gut.^55^ Although it remains unclear whether enteric‐glial‐derived IL‐6 functionally affects POI or its underlying inflammation, it has recently been shown to trigger SPP1^+^ protumorigenic tumor‐associated macrophage polarization,^56^ emphasizing its potential to shape the immune microenvironment directly. Besides IL‐1β, IL‐1α also signals via the IL‐1R1, and in enteric glia, both cytokines induce IL‐6 release to the same extent.^57^ Subsequent work with a conditional knock‐out of IL‐1R1 in glial cells (GFAP^Cre^ × IL‐1R1^fl/fl^) provided the missing piece of evidence that glial IL‐1R1 signaling contributed to POI pathology, a fact further underlined by the induction of a reactive phenotype by IL‐1R1 signaling in human enteric glia.^36^ In line with the improved gastrointestinal transit, IL‐1R1 deficient enteric glia exhibited a reduced expression of reactive glia‐related genes and reduced gliosis,^36^ a term describing an inflammatory state of glia‐harboring tissue. In the same study, a Cre‐loxP‐based technique involving the selective expression of a ribosomal protein tagged with hemagglutinin A (HA) in a target cell type^58^ (e.g., Sox‐10^iCreERT2^ × Rpl22‐HA^fl/fl^ mice), called *RiboTag*, paired with a subsequent bulk RNA‐Seq analysis uncovered enteric glial reactivity as a part of the immediate, early response in POI pathogenesis. Simultaneously, a comparable immediate reactive glial signature in POI has been observed with another *RiboTag* approach using Plp^iCreERT2^ × Rpl22‐HA^fl/fl^ mice.^40^ Although the overlap in glial signatures obtained from Sox10^+^ and Plp^+^ cells was not unexpected due to the shared expression of both markers in most glial cells, these studies complement each other and indicate that this effect occurs in the majority of glial cells in the *muscularis externa*.

In addition to IL‐6, other immunologically active molecules partake in the immediate enteric glial reactivity in POI. Interestingly, many of them belong to the class of chemoattractants (e.g., CCL2, CXCL1, CXCL2, CXCL5, CCL5, CCL7, and CXCL10).^10^, ^35^, ^36^, ^40^, ^46^, ^59^ Among these reactive enteric glia‐derived chemokines in POI, CCL2 is the best characterized as it displayed the highest induction. Current evidence suggests that glial‐derived CCL2 plays a particular role in monocyte recruitment.^39^, ^40^ Stakenborg et al. have shown that glial‐derived CCL2 increased monocyte migration in an in vitro transwell system.^40^ Furthermore, a reduced presence of ganglia‐associated, activated CD68^+^ macrophages, and diminished levels of glial‐derived CCL2 during POI in mice with glial‐restricted IL‐1R1 deficiency confirmed the idea of a chemoattractant effect of enteric glia after IL‐1 stimulation.^36^ As other cells can also release CCL2, a final in vivo proof, for example, through conditional glial CCL2 knockout mice, is needed to validate if glial‐derived CCL2 contributes to myeloid cell recruitment and to prove the exact functional changes of macrophages like their polarization.

In addition to their chemoattractive capacity, reactive glia were shown to affect resident and infiltrating macrophage function and differentiation. To this end, enteric glia not only support their recruitment by CCL2 release but also trigger the differentiation towards an anti‐inflammatory phenotype via CSF‐1.^36^, ^40^, ^41^ Importantly, pro‐resolving monocyte differentiation was suggested to be a potential therapeutic target in treating intestinal inflammation.^60^, ^61^ The impact of ENS‐released CSF‐1 on macrophage development, maintenance, and activation is not completely new. Under homeostasis, enteric neuron‐derived CSF‐1 contributes to motility patterns in a microbiota‐driven manner,^62^ with interstitial cells of Cajal (ICC) contributing to its production.^63^ A recent study suggests that within the bowel wall, even in homeostasis, ICC and enteric glia, but not enteric neurons, are the main sources of CSF‐1.^41^ In human enteric glia, a combined LPS and IFNɣ treatment induced a reactive phenotype and confirmed them as a prominent CSF‐1 producer.^45^ Additionally, another study revealed that reactive enteric glial plasticity plays a role in POI resolution^40^ via the release of CSF‐1, which can drive the recruitment and differentiation of pro‐resolving macrophages. Further identification of CSF‐1 expressing cell clusters, perhaps even one specific for enteric glia, through scRNA‐Seq studies, is required to enable the generation of conditional, cell‐restricted CSF‐1 knockout mice and ultimately elucidate the relevant molecular source of CSF‐1.

Different groups established that immune mediators like LPS,^46^ ATP,^35^ IL‐1,^36^, ^40^ and IFNɣ^10^ induce glial reactivity. Another important molecule linking glial reactivity and tissue damage is ATP. Elevated extracellular ATP concentrations, occurring during tissue damage and inflammation, induce the expression of cytokines (e.g., IL‐6), chemokines (e.g., CCL2), and GFAP.^35^ A pharmacological study revealed that P2X receptors are involved in ATP‐induced glial reactivity during POI,^35^ and the drug Ambroxol, a P2X2‐antagonist, prevented ATP‐induced enteric glial reactivity and protected mice from POI. It also abrogated enteric reactivity in human intestinal cultures exposed to mechanical trauma in vitro.^35^ The role of extracellular ATP is also relevant to enteric neurons as it induces enteric neuronal death via Panx1 and P2X7 channels.^64^ During inflammation, purine release triggers glial activation, leading to pathogenic ATP discharge by glial cells. The increased levels of extracellular ATP then cause neuronal death through the activation of neuronal P2X7 receptors.^29^ Due to the multitude of purinergic receptors, more in‐depth studies are needed to understand the complex role of purinergic signaling in glial reactivity.

Interestingly, recent observations indicate that glial reactivity can also be induced in response to sympathetic hyperactivity, occurring as a direct event in response to surgery.^37^ In Sox10^iCreERT2^ × Rpl22HA^fl/fl^ mice, a simple abdominal incision without additional manipulation of the visceral surfaces induced enteric reactivity, including gene expression related to adrenergic signaling.^37^ Optogenetic and pharmacological stimulation of β1/2‐adrenergic downstream signaling confirmed that adrenergic pathways induce enteric glial reactivity upon laparotomy.^37^ As enteric glial reactivity was diminished in animals that underwent chemical denervation prior to surgery, these data show that adrenergic signaling might be a priming event of enteric glial reactivity. Previous findings in the guinea pig colon by Gulbransen et al. indicate that ATP released from sympathetic nerves can activate enteric glial cells through critical calcium signaling pathways.^65^ Specifically, ATP is the only purinergic neurotransmitter shown to trigger such responses in enteric glia, indicating its unique role in this process.^65^

Importantly, enteric glial activation and the subsequent immune modulation is a multifactorial cascade, with several potential glial activators,^35^ resulting in the reactive glial phenotype. For POI, this activation cascade could potentially be initiated by two different routes. The first might be a passive release of IL‐1α, which has been shown to stimulate glial IL‐6 and CCL2 release to the same extent as IL‐1β.^57^ As IL‐1α is ubiquitously expressed and can be actively or passively released, for example, from dying cells after surgical trauma, it might trigger the glial activation cascade prior to IL‐1β, which mainly originates from infiltrating monocyte‐derived macrophages at later stages of the acute inflammation. The second pathway could be β1/2‐adrenergic signaling as the immediate trigger of glial reactivity after skin incision.^37^ The resulting cytokine and chemokine release by enteric glia, including CCL2, would then recruit infiltrating monocytes^66^ and, via IL‐6^67^ and other mediators,^41^ drive their differentiation into macrophages. The additional secretion of both IL‐1 ligands by infiltrating monocyte‐derived macrophages then boosts the reactive glial phenotype by enhancing the release of immune modulators.^36^, ^40^

Another example comes from a bulk RNA‐seq analysis of reactive enteric glia at later stages of POI.^37^ There, a molecular shift towards pro‐resolving gene expression signatures was observed 72 h after surgery. These observations indicate that the nature of enteric glial reactivity is, per se, neither detrimental nor beneficial but dependent on the stage of inflammation and tissue environment.

### 2.2 Reactive enteric glia in chronic inflammation

Inflammation in the gut might progress toward a chronic state due to insufficient resolution and recurring inflammatory reactions. One example is inflammatory bowel disease (IBD), which might develop due to genetic and environmental factors as well as abnormal responses of the host microbiome.^68^, ^69^, ^70^ Contemporary research has shown that enteric neuroinflammation is associated with and contributes to IBD progression, with several immune cell populations interacting with enteric glial cells.^13^ Moreover, prolonged activation of EGCs has been associated with worsening IBD symptoms, including anxiety‐ and depressive‐like behaviors.^71^

In chronic intestinal inflammation, the crosstalk between EGCs and macrophages has been the most thoroughly examined. One aspect is pro‐inflammatory stimuli, such as IL‐1β‐induced glial reactivity or signaling through Cx43 hemichannels, which ultimately activate tumor necrosis factor α converting enzyme (TACE). Glial Cx43‐dependent TACE activation then results in proteolytic cleavage of cell membrane CSF‐1 (mCSF‐1), increased release of soluble CSF‐1 (sCSF‐1), and, in turn, macrophage activation.^41^ Novel data indicates the capability of some macrophages to not only surround enteric ganglia but also infiltrate them.^43^ While these so‐called “intraganglionic” macrophages already exist under physiological conditions, their numbers increased in experimental DSS colitis, where they are implied to contribute to the degradation of the myenteric plexus barrier, thereby leaving enteric neurons and glia “unguarded” from infiltrating immune cells.^5^, ^43^ However, data about specific spatial interactions between enteric glia and macrophages, whether in the intra‐ or extra‐ganglionic regions, their location along the GI tract, or across the bowel wall layers, are largely missing.

In addition to the immunomodulatory function of Cx43/TACE signaling, activation of Cx43 in enteric glia transformed otherwise innocuous stimuli into painful signals during gut inflammation.^41^ This visceral hypersensitivity triggered enteric glial prostaglandin E2 release, subsequently activating EP4 receptors on sensory nerve terminals. Blocking Cx43 or EP4 reduced nociceptor sensitivity in response to glial stimulation, indicating that targeting enteric glial‐neuron signaling is promising for alleviating visceral pain associated with inflammatory disorders.^42^ Besides their action on visceral pain transduction, reactive enteric glia have also been shown to be involved in enteric neuronal death. Brown et al. showed that purinergic activation of enteric glia induced Ca^2+^‐responses and enteric neuron death in a 2,4‐dinitrobenzene sulfonic acid mouse model of colitis.^29^ The neurotoxic activity was related to glial Cx43 signaling and driven by NO production from glial iNOS, resulting in elevated ATP release through Cx43 hemichannels.^29^ Indeed, enteric neuronal loss is a common phenotype observed in chronic inflammation, and oxidative ENS stress^72^ might be another essential trigger. For example, one study found that oxidative‐stress‐induced HMGB‐1‐release functioned as a DAMP, triggering glial reactivity and neuronal death in DSS‐, oxazolone‐ and adoptive T‐cell‐induced colitis models.^73^

Typical markers of reactive enteric glia found in acute inflammation were also found in chronically inflamed intestines but showed different expression patterns. Gonzalez Acera et al. presented differentially expressed “neuroglial identifier genes”, previously identified by single‐cell RNA sequencing,^8^ such as *S100β*, *Gfap*, and *Sox10*, along the disease course of DSS‐induced colitis.^44^ Interestingly, when comparing murine DSS‐induced colitis models, neuronal and reactive enteric glial markers were enriched in acute colitis but dropped to baseline levels during chronic colitis.^44^ In patient samples with active ulcerative colitis (UC), neuroglial identifiers were sex‐independently expressed 38% higher compared to healthy controls, while their expression was reduced during UC remission.^44^ Furthermore, most glial and neuronal marker genes were particularly enriched in DSS‐induced colitis, with less pronounced induction in T‐cell‐adoptive transfer colitis and Oxazolon‐induced colitis, respectively. As these colitis models are based on T‐cell mediated immune responses, one might speculate that the less pronounced ENS signatures result from weaker T‐cell/ENS interactions than the well‐established ENS/monocyte/macrophage interactions. Nevertheless, some studies described potential pathways of T‐cell/ENS interactions.^74^, ^75^, ^76^, ^77^ In addition, enteric glia interact with another lymphoid cell population of the innate immune system, the so‐called innate lymphoid cells (ILC). Ibiza et al. showed that the particular ILC subtype 3 interacts with enteric glia and controls IL‐22 production and, thereby, barrier defense.^78^

These data indicate that acute enteric glial reactivity might not be sustained within chronic stages. However, reactive glia might switch to a different molecular phenotype during the chronic state of the disease or become less sensitive and thus reactive to repetitive inflammatory conditions. Longitudinal patient biopsy collections offer an avenue to explore the role of enteric glial reactivity and plasticity in such chronic conditions.

Aggravating the complex role of enteric glia in chronic inflammation, a recent study showed that gut‐affecting chronic stress links the CNS and extrinsic innervation, thereby controlling neuronal dysfunction and inflammation‐promoting enteric glial reactivity.^79^ Interestingly, Schneider et al. claim that reactive enteric glia‐derived CSF‐1, which induces the differentiation of pro‐resolving monocyte‐derived macrophages in POI, promotes a pro‐inflammatory phenotype of macrophages during colitis.

Subsumed, the current literature indicates a rather detrimental role of reactive glia in chronic inflammation, while studies showing beneficial effects are scarce. Functionally, reactive enteric glia control distinct immune mediators released in chronic inflammation, impair associated visceral pain, and increase neuronal death, while their effect on the overall inflammation state and disease outcome remains unclear.

## 3 CONCLUSION AND OPEN QUESTIONS

Enteric glial reactivity has been proven to be part of and mechanistically linked to acute and chronic intestinal inflammation. Several studies confirmed enteric glial plasticity during inflammation and documented several mediators, including ATP, IL‐1, LPS, IFNɣ, and NE, inducing the release of immunoactive mediators from reactive glia. While this supports a central role of enteric glia in intestinal inflammation, the following aspects require further attention.

During acute inflammation, the reactive glial response seems to induce common but also stimulus‐selective molecular reactivity patterns. Investigation of a disease‐independent core signature in reactive enteric glia and its changes from initiation to resolution will be worthwhile. In case reactivity states can be correlated with distinct inflammatory phases, studies into potential biomarkers in biological fluids or stool will be tremendously helpful in assessing disease states and progression or the responsiveness to medications. Moreover, differences between the acute and chronic inflammatory states and their impact on enteric glia reactivity have to be further investigated. While most transcriptionally obtained mediators and functionally verified cytokines overlap between acute and chronic inflammation, their modes of operation can differ greatly. These differences might originate from repeated insults during a chronic model and subsequently altered capacity of enteric glia, similar to astrocytes that received multiple insults.^80^ Additionally, the overall microenvironment changes drastically during multiple relapsing–remitting phases of chronic inflammation, thereby altering the function of key mediators such as IL‐6,^81^ CCL2,^82^ and sympathetic neurotransmitters.^83^ Moreover, interactions between enteric glia and other resident or infiltrating immune cells might change throughout different inflammatory states. The intercellular communication between enteric glia and immune cells has been primarily studied during acute inflammation,^40^, ^60^, ^61^ while its effects in chronic disease states are less well understood and largely related to cancer studies^56^ or focus on the involvement rather than the interplay of these cell types.^56^

Given the plasticity of enteric glia during the inflammatory course and the recently identified existence of transcriptionally different enteric glial subpopulations in humans and mice,^6^, ^8^ it will be important to identify and characterize disease‐specific reactive glia subpopulations by single‐cell or single‐nuclei sequencing. Moreover, spatial transcriptomics might help identify novel intercellular communication, for example, between distinct glia subtypes and specific immune cell populations, like intraganglionic and extraganglionic macrophages. Comprehensive single‐nuclei or spatial transcriptomics from longitudinally collected surgical and endoscopic samples will help decipher glial reactivity in the different disease stages of patients. However, as it remains unclear whether the transcriptional profile of enteric glia, especially during inflammation, is stable, these transcriptomic analyses must be validated by functional studies and proteomic or histological methods. Even extensive scRNA‐Seq studies^8^, ^9^, ^10^ only showed a few clusters of homeostatic and reactive enteric glia, so additional studies are needed to elucidate specific subtypes and their involvement during distinct diseases. Moreover, similar methods have to be employed to verify the functional relevance of these transcripts in the context of the respective tissue state.

## AUTHOR CONTRIBUTIONS

RS, LS, EH, PL, and SW contributed to conceptualization, original drafting, writing, reviewing and editing, literature search, and illustrations.

## CONFLICT OF INTEREST STATEMENT

No conflicts of interest to disclose.

## Data Availability

Data sharing is not applicable to this article as no new data were created or analyzed in this study.

## References

[1] Seguella L , Gulbransen BD . Enteric glial biology, intercellular signalling and roles in gastrointestinal disease. Nat Rev Gastroenterol Hepatol. 2021;18(8):571‐587. doi:10.1038/s41575-021-00423-7 33731961 PMC8324524

[2] Bubeck M , Becker C , Patankar JV . Guardians of the gut: influence of the enteric nervous system on the intestinal epithelial barrier. Front Med (Lausanne). 2023;10:1228938. doi:10.3389/fmed.2023.1228938 37692784 PMC10485265

[3] Rao M , Rastelli D , Dong L , et al. Enteric glia regulate gastrointestinal motility but are not required for maintenance of the epithelium in mice. Gastroenterology. 2017;153(4):1068‐1081.e7. doi:10.1053/j.gastro.2017.07.002 28711628 PMC5623141

[4] Savidge TC , Newman P , Pothoulakis C , et al. Enteric glia regulate intestinal barrier function and inflammation via release of S‐nitrosoglutathione. Gastroenterology. 2007;132(4):1344‐1358. doi:10.1053/j.gastro.2007.01.051 17408650

[5] Vergnolle N , Cirillo C . Neurons and glia in the enteric nervous system and epithelial barrier function. Physiology (Bethesda). 2018;33(4):269‐280. doi:10.1152/physiol.00009.2018 29897300 PMC6088142

[6] Laddach A , Chng SH , Lasrado R , et al. A branching model of lineage differentiation underpinning the neurogenic potential of enteric glia. Nat Commun. 2023;14(1):5904. doi:10.1038/s41467-023-41492-3 37737269 PMC10516949

[7] Seguella L , McClain JL , Esposito G , Gulbransen BD . Functional intraregional and interregional heterogeneity between myenteric glial cells of the colon and Duodenum in mice. J Neurosci. 2022;42(46):8694‐8708. doi:10.1523/JNEUROSCI.2379-20.2022 36319118 PMC9671584

[8] Drokhlyansky E , Smillie CS , van Wittenberghe N , et al. The human and mouse enteric nervous system at single‐cell resolution. Cell. 2020;182(6):1606‐1622.e23. doi:10.1016/j.cell.2020.08.003 32888429 PMC8358727

[9] Zeisel A , Hochgerner H , Lönnerberg P , et al. Molecular architecture of the mouse nervous system. Cell. 2018;174(4):999‐1014.e22. doi:10.1016/j.cell.2018.06.021 30096314 PMC6086934

[10] Progatzky F , Shapiro M , Chng SH , et al. Regulation of intestinal immunity and tissue repair by enteric glia. Nature. 2021;599(7883):125‐130. doi:10.1038/s41586-021-04006-z 34671159 PMC7612231

[11] Santhosh S , Zanoletti L , Stamp LA , Hao MM , Matteoli G . From diversity to disease: unravelling the role of enteric glial cells. Front Immunol. 2024;15:1408744. doi:10.3389/fimmu.2024.1408744 38957473 PMC11217337

[12] Grubišić V , Gulbransen BD . Enteric glia: the most alimentary of all glia. J Physiol. 2017;595(2):557‐570. doi:10.1113/JP271021 27106597 PMC5233670

[13] Le Berre C , Naveilhan P , Rolli‐Derkinderen M . Enteric glia at center stage of inflammatory bowel disease. Neurosci Lett. 2023;809:137315. doi:10.1016/j.neulet.2023.137315 37257681

[14] Boesmans W , Lasrado R , Vanden Berghe P , Pachnis V . Heterogeneity and phenotypic plasticity of glial cells in the mammalian enteric nervous system. Glia. 2015;63(2):229‐241. doi:10.1002/glia.22746 25161129

[15] Scavuzzo MA , Letai KC , Maeno‐Hikichi Y , et al. Enteric glial hub cells coordinate intestinal motility. *bioRxiv* . 2023. doi:10.1101/2023.06.07.544052

[16] Thomasi B , Gulbransen B . Mini‐review: intercellular communication between enteric glia and neurons. Neurosci Lett. 2023;806:137263. doi:10.1016/j.neulet.2023.137263 37085112 PMC10150911

[17] Sharkey KA , Mawe GM . The enteric nervous system. Physiol Rev. 2023;103(2):1487‐1564. doi:10.1152/physrev.00018.2022 36521049 PMC9970663

[18] Michel K , Kuch B , Dengler S , Demir IE , Zeller F , Schemann M . How big is the little brain in the gut? Neuronal numbers in the enteric nervous system of mice, Guinea pig, and human. Neurogastroenterol Motil. 2022;34(12):e14440. doi:10.1111/nmo.14440 35929768

[19] Hoff S , Zeller F , von Weyhern CWH , et al. Quantitative assessment of glial cells in the human and Guinea pig enteric nervous system with an anti‐Sox8/9/10 antibody. J Comp Neurol. 2008;509(4):356‐371. doi:10.1002/cne.21769 18512230

[20] D'Errico F , Goverse G , Dai Y , et al. Estrogen receptor β controls proliferation of enteric glia and differentiation of neurons in the myenteric plexus after damage. Proc Natl Acad Sci USA. 2018;115(22):5798‐5803. doi:10.1073/pnas.1720267115 29760072 PMC5984503

[21] Belkind‐Gerson J , Graham HK , Reynolds J , et al. Colitis promotes neuronal differentiation of Sox2+ and PLP1+ enteric cells. Sci Rep. 2017;7(1):2525. doi:10.1038/s41598-017-02890-y 28566702 PMC5451421

[22] Laranjeira C , Sandgren K , Kessaris N , et al. Glial cells in the mouse enteric nervous system can undergo neurogenesis in response to injury. J Clin Invest. 2011;121(9):3412‐3424. doi:10.1172/JCI58200 21865647 PMC3163972

[23] Linan‐Rico A , Ochoa‐Cortes F , Schneider R , Christofi FL . Mini‐review: enteric glial cell reactions to inflammation and potential therapeutic implications for GI diseases, motility disorders, and abdominal pain. Neurosci Lett. 2023;812:137395. doi:10.1016/j.neulet.2023.137395 37451357 PMC10952371

[24] Tani G , Tomuschat C , O'Donnell AM , Coyle D , Puri P . Increased population of immature enteric glial cells in the resected proximal ganglionic bowel of Hirschsprung's disease patients. J Surg Res. 2017;218:150‐155. doi:10.1016/j.jss.2017.05.062 28985842

[25] Sunardi M , Cirillo C . Mini‐review: "enteric glia functions in nervous tissue repair: therapeutic target or tool?". Neurosci Lett. 2023;812:137360. doi:10.1016/j.neulet.2023.137360 37393007

[26] Baghdadi MB , Ayyaz A , Coquenlorge S , et al. Enteric glial cell heterogeneity regulates intestinal stem cell niches. Cell Stem Cell. 2022;29(1):86‐100.e6. doi:10.1016/j.stem.2021.10.004 34727519

[27] Progatzky F , Pachnis V . The role of enteric glia in intestinal immunity. Curr Opin Immunol. 2022;77:102183. doi:10.1016/j.coi.2022.102183 35533467 PMC9586875

[28] Boesmans W , Nash A , Tasnády KR , Yang W , Stamp LA , Hao MM . Development, diversity, and neurogenic capacity of enteric glia. Front Cell Dev Biol. 2021;9:775102. doi:10.3389/fcell.2021.775102 35111752 PMC8801887

[29] Brown IAM , McClain JL , Watson RE , Patel BA , Gulbransen BD . Enteric glia mediate neuron death in colitis through purinergic pathways that require connexin‐43 and nitric oxide. Cell Mol Gastroenterol Hepatol. 2016;2(1):77‐91. doi:10.1016/j.jcmgh.2015.08.007 26771001 PMC4707972

[30] Delvalle NM , Dharshika C , Morales‐Soto W , Fried DE , Gaudette L , Gulbransen BD . Communication between enteric neurons, glia, and nociceptors underlies the effects of tachykinins on Neuroinflammation. Cell Mol Gastroenterol Hepatol. 2018;6(3):321‐344. doi:10.1016/j.jcmgh.2018.05.009 30116771 PMC6091443

[31] McClain JL , Fried DE , Gulbransen BD . Agonist‐evoked Ca2+ signaling in enteric glia drives neural programs that regulate intestinal motility in mice. Cell Mol Gastroenterol Hepatol. 2015;1(6):631‐645. doi:10.1016/j.jcmgh.2015.08.004 26693173 PMC4673674

[32] Brown IAM , Gulbransen BD . The antioxidant glutathione protects against enteric neuron death in situ, but its depletion is protective during colitis. Am J Physiol Gastrointest Liver Physiol. 2018;314(1):G39‐G52. doi:10.1152/ajpgi.00165.2017 28882823 PMC5866372

[33] Abdo H , Derkinderen P , Gomes P , et al. Enteric glial cells protect neurons from oxidative stress in part via reduced glutathione. FASEB J. 2010;24(4):1082‐1094. doi:10.1096/fj.09-139519 19906678

[34] Grubišić V , Perez‐Medina AL , Fried DE , et al. NTPDase1 and −2 are expressed by distinct cellular compartments in the mouse colon and differentially impact colonic physiology and function after DSS colitis. Am J Physiol Gastrointest Liver Physiol. 2019;317(3):G314‐G332. doi:10.1152/ajpgi.00104.2019 31188623 PMC6774087

[35] Schneider R , Leven P , Glowka T , et al. A novel P2X2‐dependent purinergic mechanism of enteric gliosis in intestinal inflammation. EMBO Mol Med. 2021;13(1):e12724. doi:10.15252/emmm.202012724 33332729 PMC7799361

[36] Schneider R , Leven P , Mallesh S , et al. IL‐1‐dependent enteric gliosis guides intestinal inflammation and dysmotility and modulates macrophage function. Commun Biol. 2022;5(1):811. doi:10.1038/s42003-022-03772-4 35962064 PMC9374731

[37] Leven P , Schneider R , Schneider L , et al. β‐Adrenergic signaling triggers enteric glial reactivity and acute enteric gliosis during surgery. J Neuroinflammation. 2023;20(1):255. doi:10.1186/s12974-023-02937-0 37941007 PMC10631040

[38] Mazzotta E , Grants I , Villalobos‐Hernandez E , et al. BQ788 reveals glial ETB receptor modulation of neuronal cholinergic and nitrergic pathways to inhibit intestinal motility: linked to postoperative ileus. Br J Pharmacol. 2023;180(19):2550‐2576. doi:10.1111/bph.16145 37198101 PMC11085045

[39] Stoffels B , Hupa KJ , Snoek SA , et al. Postoperative ileus involves interleukin‐1 receptor signaling in enteric glia. Gastroenterology. 2014;146(1):176‐187.e1. doi:10.1053/j.gastro.2013.09.030 24067878

[40] Stakenborg M , Abdurahiman S , de Simone V , et al. Enteric glial cells favor accumulation of anti‐inflammatory macrophages during the resolution of muscularis inflammation. Mucosal Immunol. 2022;15(6):1296‐1308. doi:10.1038/s41385-022-00563-2 36071145 PMC9705256

[41] Grubišić V , McClain JL , Fried DE , et al. Enteric glia modulate macrophage phenotype and visceral sensitivity following inflammation. Cell Rep. 2020;32(10):108100. doi:10.1016/j.celrep.2020.108100 32905782 PMC7518300

[42] Morales‐Soto W , Gonzales J , Jackson WF , Gulbransen BD . Enteric glia promote visceral hypersensitivity during inflammation through intercellular signaling with gut nociceptors. Sci Signal. 2023;16(812):eadg1668. doi:10.1126/scisignal.adg1668 37988454 PMC10733972

[43] Dora D , Ferenczi S , Stavely R , et al. Evidence of a myenteric plexus barrier and its macrophage‐dependent degradation during murine colitis: implications in enteric Neuroinflammation. Cell Mol Gastroenterol Hepatol. 2021;12(5):1617‐1641. doi:10.1016/j.jcmgh.2021.07.003 34246810 PMC8551790

[44] Gonzalez Acera M , Bubeck M , Mascia F , et al. Dynamic, transient, and robust increase in the innervation of the inflamed mucosa in inflammatory bowel diseases. Cells. 2021;10(9):2253. doi:10.3390/cells10092253 34571902 PMC8471820

[45] Liñán‐Rico A , Turco F , Ochoa‐Cortes F , et al. Molecular signaling and dysfunction of the human reactive enteric glial cell phenotype: implications for GI infection, IBD, POI, neurological, motility, and GI disorders. Inflamm Bowel Dis. 2016;22(8):1812‐1834. doi:10.1097/MIB.0000000000000854 27416040 PMC4993196

[46] Rosenbaum C , Schick MA , Wollborn J , et al. Activation of myenteric glia during acute inflammation in vitro and in vivo. PLoS One. 2016;11(3):e0151335. doi:10.1371/journal.pone.0151335 26964064 PMC4786261

[47] Ahmadzai MM , Seguella L , Gulbransen BD . Circuit‐specific enteric glia regulate intestinal motor neurocircuits. Proc Natl Acad Sci USA. 2021;118(40):e2025938118. doi:10.1073/pnas.2025938118 34593632 PMC8501758

[48] Kovler ML , Gonzalez Salazar AJ , Fulton WB , et al. Toll‐like receptor 4‐mediated enteric glia loss is critical for the development of necrotizing enterocolitis. Sci Transl Med. 2021;13(612):eabg3459. doi:10.1126/scitranslmed.abg3459 34550727 PMC8859973

[49] Delvalle NM , Fried DE , Rivera‐Lopez G , Gaudette L , Gulbransen BD . Cholinergic activation of enteric glia is a physiological mechanism that contributes to the regulation of gastrointestinal motility. Am J Physiol Gastrointest Liver Physiol. 2018;315(4):G473‐G483. doi:10.1152/ajpgi.00155.2018 29927320 PMC6230698

[50] McClain J , Grubišić V , Fried D , et al. Ca2+ responses in enteric glia are mediated by connexin‐43 hemichannels and modulate colonic transit in mice. Gastroenterology. 2014;146(2):497‐507.e1. doi:10.1053/j.gastro.2013.10.061 24211490 PMC3935238

[51] Boesmans W , Hao MM , Fung C , et al. Structurally defined signaling in neuro‐glia units in the enteric nervous system. Glia. 2019;67(6):1167‐1178. doi:10.1002/glia.23596 30730592 PMC6593736

[52] Ahmadzai MM , McClain JL , Dharshika C , et al. LPAR1 regulates enteric nervous system function through glial signaling and contributes to chronic intestinal pseudo‐obstruction. J Clin Invest. 2022;132(4):6079. doi:10.1172/JCI149464 PMC8843750 35166239

[53] Gulbransen BD , Christofi FL . Are we close to targeting enteric glia in gastrointestinal diseases and motility disorders? Gastroenterology. 2018;155(2):245‐251. doi:10.1053/j.gastro.2018.06.050 29964042 PMC6452442

[54] van Bree SHW , Nemethova A , Cailotto C , Gomez‐Pinilla PJ , Matteoli G , Boeckxstaens GE . New therapeutic strategies for postoperative ileus. Nat Rev Gastroenterol Hepatol. 2012;9(11):675‐683. doi:10.1038/nrgastro.2012.134 22801725

[55] Chiou S‐H , Tseng D , Reuben A , et al. Global analysis of shared T cell specificities in human non‐small cell lung cancer enables HLA inference and antigen discovery. Immunity. 2021;54(3):586‐602.e8. doi:10.1016/j.immuni.2021.02.014 33691136 PMC7960510

[56] van Baarle L , De Simone V , Schneider L , et al. IL‐1R signaling drives enteric glia‐macrophage interactions in colorectal cancer. Nat Commun. 2024;15(1):6079. doi:10.1038/s41467-024-50438-2 39030280 PMC11271635

[57] Hupa KJ , Stein K , Schneider R , et al. AIM2 inflammasome‐derived IL‐1β induces postoperative ileus in mice. Sci Rep. 2019;9(1):10602. doi:10.1038/s41598-019-46968-1 31332247 PMC6646358

[58] Leven P , Schneider R , Siemens KD , Jackson WS , Wehner S . Application of a RiboTag‐based approach to generate and analyze mRNA from enteric neural cells. Neurogastroenterol Motil. 2022;34(7):e14309. doi:10.1111/nmo.14309 34939271

[59] Grubišić V , Bali V , Fried DE , et al. Enteric glial adenosine 2B receptor signaling mediates persistent epithelial barrier dysfunction following acute DSS colitis. Mucosal Immunol. 2022;15(5):964‐976. doi:10.1038/s41385-022-00550-7 35869148 PMC9385475

[60] Viola MF , Chavero‐Pieres M , Modave E , et al. Dedicated macrophages organize and maintain the enteric nervous system. Nature. 2023;618(7966):818‐826. doi:10.1038/s41586-023-06200-7 37316669

[61] Delfini M , Stakenborg N , Viola MF , Boeckxstaens G . Macrophages in the gut: masters in multitasking. Immunity. 2022;55(9):1530‐1548. doi:10.1016/j.immuni.2022.08.005 36103851

[62] Muller PA , Koscsó B , Rajani GM , et al. Crosstalk between muscularis macrophages and enteric neurons regulates gastrointestinal motility. Cell. 2014;158(2):300‐313. doi:10.1016/j.cell.2014.04.050 25036630 PMC4149228

[63] Breland A , Ha SE , Jorgensen BG , et al. Smooth muscle transcriptome browser: offering genome‐wide references and expression profiles of transcripts expressed in intestinal SMC, ICC, and PDGFRα+ cells. Sci Rep. 2019;9(1):387. doi:10.1038/s41598-018-36607-6 30674925 PMC6344548

[64] Gulbransen BD , Bashashati M , Hirota SA , et al. Activation of neuronal P2X7 receptor‐pannexin‐1 mediates death of enteric neurons during colitis. Nat Med. 2012;18(4):600‐604. doi:10.1038/nm.2679 22426419 PMC3321107

[65] Gulbransen BD , Bains JS , Sharkey KA . Enteric glia are targets of the sympathetic innervation of the myenteric plexus in the Guinea pig distal colon. J Neurosci. 2010;30(19):6801‐6809. doi:10.1523/JNEUROSCI.0603-10.2010 20463242 PMC6632550

[66] McClellan JL , Davis JM , Steiner JL , et al. Linking tumor‐associated macrophages, inflammation, and intestinal tumorigenesis: role of MCP‐1. Am J Physiol Gastrointest Liver Physiol. 2012;303(10):G1087‐G1095. doi:10.1152/ajpgi.00252.2012 23019193 PMC3517651

[67] Chomarat P , Banchereau J , Davoust J , Palucka AK . IL‐6 switches the differentiation of monocytes from dendritic cells to macrophages. Nat Immunol. 2000;1(6):510‐514. doi:10.1038/82763 11101873

[68] Guan Q . A comprehensive review and update on the pathogenesis of inflammatory bowel disease. J Immunol Res. 2019;2019:7247238. doi:10.1155/2019/7247238 31886308 PMC6914932

[69] Rogler G , Vavricka S . Exposome in IBD: recent insights in environmental factors that influence the onset and course of IBD. Inflamm Bowel Dis. 2015;21(2):400‐408. doi:10.1097/MIB.0000000000000229 25358064

[70] Cleynen I , Boucher G , Jostins L , et al. Inherited determinants of Crohn's disease and ulcerative colitis phenotypes: a genetic association study. Lancet. 2016;387(10014):156‐167. doi:10.1016/S0140-6736(15)00465-1 26490195 PMC4714968

[71] Li Y , Wang Y , Sun Q , et al. Inhibiting the activation of enteric glial cells alleviates intestinal inflammation and comorbid anxiety‐ and depressive‐like behaviors in the ulcerative colitis mice. Neurochem Int. 2024;178:105789. doi:10.1016/j.neuint.2024.105789 38852824

[72] Stavely R , Ott LC , Rashidi N , Sakkal S , Nurgali K . The oxidative stress and nervous distress connection in gastrointestinal disorders. Biomolecules. 2023;13(11):1586. doi:10.3390/biom13111586 38002268 PMC10669114

[73] Stavely R , Sahakian L , Filippone RT , et al. Oxidative stress‐induced HMGB1 translocation in myenteric neurons contributes to neuropathy in colitis. Biomolecules. 2022;12(12):1831. doi:10.3390/biom12121831 36551259 PMC9776169

[74] Chandrasekharan B , Nezami BG , Srinivasan S . Emerging neuropeptide targets in inflammation: NPY and VIP. Am J Physiol Gastrointest Liver Physiol. 2013;304(11):G949‐G957. doi:10.1152/ajpgi.00493.2012 23538492 PMC3680683

[75] Yan Y , Ramanan D , Rozenberg M , et al. Interleukin‐6 produced by enteric neurons regulates the number and phenotype of microbe‐responsive regulatory T cells in the gut. Immunity. 2021;54(3):499‐513.e5. doi:10.1016/j.immuni.2021.02.002 33691135 PMC8133394

[76] Pabois J , Durand T , Le Berre C , et al. T cells show preferential adhesion to enteric neural cells in culture and are close to neural cells in the myenteric ganglia of Crohn's patients. J Neuroimmunol. 2020;349:577422. doi:10.1016/j.jneuroim.2020.577422 33068972

[77] Kermarrec L , Durand T , Neunlist M , Naveilhan P , Neveu I . Enteric glial cells have specific immunosuppressive properties. J Neuroimmunol. 2016;295‐296:79‐83. doi:10.1016/j.jneuroim.2016.04.011 27235353

[78] Ibiza S , García‐Cassani B , Ribeiro H , et al. Glial‐cell‐derived neuroregulators control type 3 innate lymphoid cells and gut defence. Nature. 2016;535(7612):440‐443. doi:10.1038/nature18644 27409807 PMC4962913

[79] Schneider KM , Blank N , Alvarez Y , et al. The enteric nervous system relays psychological stress to intestinal inflammation. Cell. 2023;186(13):2823‐2838.e20. doi:10.1016/j.cell.2023.05.001 37236193 PMC10330875

[80] Lange Canhos L , Chen M , Falk S , et al. Repetitive injury and absence of monocytes promote astrocyte self‐renewal and neurological recovery. Glia. 2021;69(1):165‐181. doi:10.1002/glia.23893 32744730

[81] Kummer KK , Zeidler M , Kalpachidou T , Kress M . Role of IL‐6 in the regulation of neuronal development, survival and function. Cytokine. 2021;144:155582. doi:10.1016/j.cyto.2021.155582 34058569

[82] Yuan J . CCR2: a characteristic chemokine receptor in normal and pathological intestine. Cytokine. 2023;169:156292. doi:10.1016/j.cyto.2023.156292 37437448

[83] Ren W , Hua M , Cao F , Zeng W . The sympathetic‐immune milieu in metabolic health and diseases: insights from pancreas, liver, intestine, and adipose tissues. Adv Sci (Weinh). 2024;11(8):e2306128. doi:10.1002/advs.202306128 38039489 PMC10885671

